# Bilateral Diffuse Uveal Melanocytic Proliferation in a Patient with Chronic Myelomonocytic Leukemia: A Rare Case and Literature Review

**DOI:** 10.3390/reports8030114

**Published:** 2025-07-19

**Authors:** Dolika D. Vasović, Miodrag Lj. Karamarković, Miroslav Jeremić, Dejan M. Rašić

**Affiliations:** 1 University Clinical Centre of Serbia, Eye Hospital, 11000 Belgrade, Serbia; 2 Faculty of Medicine, University of Belgrade, 11000 Belgrade, Serbia

**Keywords:** bilateral diffuse uveal melanocytic proliferation, paraneoplastic syndrome, chronic myelomonocytic leukemia, vision loss

## Abstract

Bilateral diffuse uveal melanocytic proliferation (BDUMP) is a rare paraneoplastic syndrome characterized by bilateral uveal melanocyte proliferation and progressive visual disturbance. While most commonly associated with solid tumors, its occurrence in hematologic malignancies is exceedingly rare. We report a case of BDUMP in a 64-year-old male recently diagnosed with chronic myelomonocytic leukemia (CMML), who presented with subacute, painless bilateral blurred vision. Multimodal imaging revealed suggestive features of BDUMP, including orange-red subretinal patches, retinal pigment epithelium mottling, and diffuse choroidal thickening, consistent with early structural involvement despite preserved central vision. No intraocular mass or signs of inflammation were observed. The patient did not receive specific treatment for BDUMP, and visual acuity remained stable during follow-up. This case underscores the importance of considering BDUMP in the differential diagnosis of bilateral visual symptoms in patients with hematologic malignancies. Although rare, BDUMP may occur in the context of CMML. Recognition through multimodal imaging and interdisciplinary collaboration is essential, and further research is needed to clarify its pathogenesis and improve management strategies.

## 1. Introduction

Bilateral diffuse uveal melanocytic proliferation (BDUMP) is a rare paraneoplastic syndrome characterized by bilateral proliferation of melanocytes within the uveal tract, often resulting in progressive visual decline. It is most frequently associated with systemic malignancies, particularly carcinomas of the lung, pancreas, and female reproductive organs [1,2]. Associations with hematologic malignancies such as chronic myelomonocytic leukemia (CMML) are exceedingly rare, with only a limited number of cases documented in the literature [1,3].

The early recognition of BDUMP is essential, as ocular manifestations may precede, coincide with, or follow the diagnosis of an underlying malignancy. Characteristic ophthalmic findings can serve as critical diagnostic clues, especially in patients without a known history of cancer, prompting timely systemic evaluation [3].

The underlying pathophysiology is believed to involve tumor-secreted circulating factors that stimulate diffuse melanocytic proliferation within the choroid, ciliary body, and iris [4]. These changes are reactive and non-neoplastic, typically without the formation of a discrete mass. Diagnosis relies on a combination of clinical signs and multimodal imaging findings. Key features include multiple pigmented and amelanotic uveal lesions, diffuse uveal thickening, exudative retinal detachment, and orange-red subretinal patches. Imaging modalities such as fluorescein angiography (FA) and fundus autofluorescence typically demonstrate the early hyperfluorescence of these lesions along with the widespread disruption of the retinal pigment epithelium (RPE) [5,6].

Despite advances in oncologic therapies, the visual prognosis in BDUMP remains poor. Management is primarily focused on treating the underlying malignancy. Immunomodulatory approaches, including plasmapheresis, intravenous immunoglobulin (IVIG), and corticosteroids, have been explored with limited and usually short-term efficacy [7].

This rare paraneoplastic entity remains underrecognized, particularly in hematologic malignancies such as CMML. Greater clinical awareness and interdisciplinary collaboration are crucial for early diagnosis and appropriate patient management.

## 2. Case Report

A 64-year-old male presented with a three-month history of progressive, painless bilateral visual disturbance. He had previously experienced clear vision, but gradually developed blurred vision that affected his ability to read. Despite a best-corrected visual acuity (BCVA) of 20/25 in both eyes, he reported subjective difficulty with fine visual tasks. He denied any history of ocular trauma, infections, or autoimmune conditions. He had been diagnosed with CMML in January 2024, approximately one month prior to the onset of visual symptoms. At the time of presentation in May 2024, he was not receiving disease-specific therapy for CMML but was undergoing supportive treatment for anemia and thrombocytopenia. Systemic staging, including contrast-enhanced CT scans of the thorax, abdomen, pelvis, and endocranium, revealedno evidence of additional malignancies.

**Figure 1:**
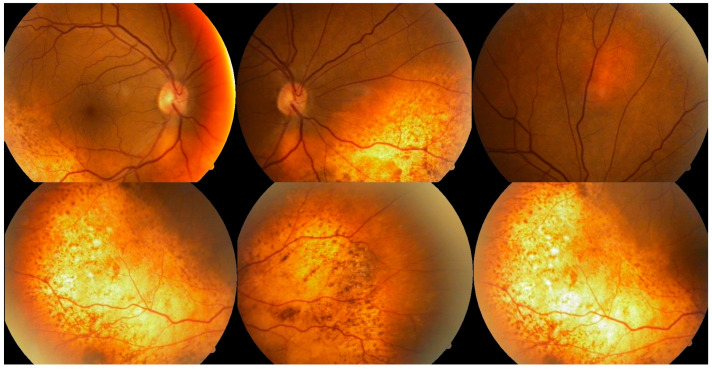
Color fundus photographs of both eyes demonstrating suggestive features of BDUMP in a 64-year-old patient with chronic myelomonocytic leukemia. **Top row** (posterior pole and optic disc views): Multiple ill-defined orange-red subretinal patches are observed, predominantly in the peripapillary and inferior posterior pole regions. RPE mottling is evident, with preserved foveal contour corresponding to stable central vision. **Bottom row** (mid-periphery and inferior retina): Subtle RPE irregularities are visible, including areas of pigment clumping and scattered lipofuscin-like deposits. Diffuse choroidal thickening and subretinal alterations contribute to the early ‘leopard-spot’ pattern suggestive of BDUMP.

Ophthalmologic evaluation revealed normal intraocular pressure in both eyes. An anterior segment examination was unremarkable, with no signs of inflammation. A dilated fundus examination showed multiple ill-defined pigmented choroidal lesions, areas of retinal pigment epithelium mottling, and diffuse uveal thickening in both eyes (Figure 1). Distinctive orange-red subretinal patches were observed bilaterally, predominantly in the posterior pole and peripapillary regions. No intraocular mass or signs of active inflammation were noted. Both lenses were clear at presentation, with no signs of cataract formation noted on slit-lamp examination.

**Figure 2:**
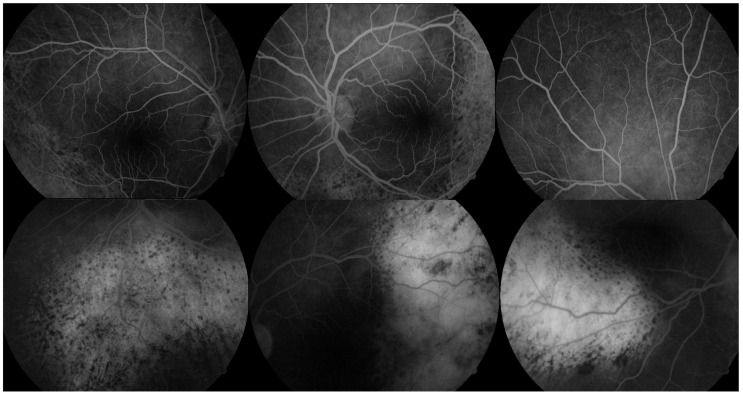
FA of both eyes illustrating vascular and RPE alterations suggestive of BDUMP. **Top row** (early-phase angiograms): Early-phase FA images show intact retinal vasculature with multiple areas of early hyperfluorescence corresponding to the orange-red subretinal lesions seen in fundus photographs. The hyperfluorescence is mottled and geographic, primarily involving the posterior pole and inferior retina. **Bottom row** (late-phase angiograms): Late-phase FA demonstrates persistent hyperfluorescence and focal pooling of dye in areas of RPE disturbance and shallow subretinal fluid. Scattered hypofluorescent zones may represent melanocytic proliferation or focal choroidal thickening, consistent with early BDUMP-related structural changes.

FA revealed multiple areas of early hyperfluorescence corresponding to the orange-red subretinal lesions, while the retinal vasculature remained largely preserved. In the late phase, persistent hyperfluorescence and focal dye pooling were noted, suggestive of RPE disruption and localized serous retinal detachment (Figure 2).

The clinical and angiographic findings supported a diagnosis of BDUMP. Given the patient’s recent diagnosis of CMML, a paraneoplastic etiology was considered highly likely. Although choroidal leukemic infiltration was included in the differential diagnosis, the bilateral and symmetric pattern, absence of mass lesions or intraocular inflammation, and the presence of features suggestive of BDUMP on multimodal imaging favored a paraneoplastic melanocytic proliferation. A biopsy was not performed, as the patient maintained good visual acuity, demonstrated no signs of ocular or systemic progression during follow-up, and the imaging findings were suggestive for BDUMP. A multidisciplinary care approach involving hematology and ophthalmology was initiated.

The patient was monitored regularly over a ten-month period, from May 2024 to March 2025. Despite close follow-up, his best-corrected visual acuity remained stable at 20/25 in both eyes. Subjectively, he continued to report mild blurring, particularly when reading or in dim lighting. Follow-up examinations revealed persistent but non-progressive subretinal fluid and stable fundoscopic findings. His CMML remained in the chronic phase, with no proliferative features and minimal systemic symptoms. Given the absence of significant visual deterioration and the patient’s hematologic stability, no systemic therapy, such as plasmapheresis or immunomodulatory treatment, was initiated. Interestingly, despite the reported rapid visual deterioration in many BDUMP cases, our patient maintained stable visual acuity over a ten-month period, suggesting a more indolent course in the context of chronic-phase CMML.

## 3. Discussion

BDUMP is an uncommon paraneoplastic syndrome characterized by bilateral melanocytic proliferation within the uveal tract. It is most frequently associated with carcinomas of the ovary, pancreas, uterus, and lung [1,2]. However, hematologic malignancies have also been rarely implicated. Cases involving B-cell lymphoma [3], myelodysplastic syndrome, and chronic leukemias have been reported, suggesting a broader neoplastic association than initially recognized [5,7].

The pathophysiological mechanism is presumed to involve circulating tumor-derived factors, such as cultured melanocyte elongation and proliferation factor (CMEPF), found in the IgG fraction of patient serum. This factor has been shown to stimulate melanocytic proliferation and disrupt the outer retina and RPE [5,7]. CMEPF may explain the diffuse uveal thickening and serous retinal detachment observed in BDUMP. These paraneoplastic changes help distinguish BDUMP from direct ocular metastases, which involve tumor cell infiltration of ocular tissues. They also differ from leukemic ocular infiltration, which typically presents with unilateral or asymmetric involvement, hemorrhage, mass-like lesions, or intraocular inflammation—features not seen in this case. Clinically, BDUMP is characterized by the presence of multiple pigmented and amelanotic uveal lesions, orange-red subretinal patches, and exudative retinal detachment. Fundus autofluorescence and FA often show early hyperfluorescence, dye pooling, and areas of RPE mottling [6,8]. Gass et al. proposed five cardinal features: (1) multiple, round or oval red-orange patches at the level of the RPE, (2) multifocal early hyperfluorescence on FA, (3) diffuse uveal thickening, (4) exudative retinal detachment, and (5) rapid cataract development [1]. Our case met several of these criteria, supporting a clinical impression suggestive of BDUMP, although definitive imaging and histopathological confirmation were not available. Although histopathological confirmation was not performed, the bilateral and symmetric pattern of imaging findings, combined with the absence of intraocular mass lesions or inflammation, was suggestive of BDUMP. The diagnosis was based on clinical observation and fluorescein angiography, interpreted in the context of an underlying hematologic malignancy. We acknowledge the lack of histopathological confirmation as a limitation of this report.

Treatment remains a challenge. While systemic therapy targeting the underlying malignancy is typically prioritized, ocular improvement is rarely achieved. Immunomodulatory approaches such as corticosteroids, intravenous immunoglobulin (IVIG), and plasmapheresis have yielded variable outcomes [5,7]. In our case, the patient did not receive any specific treatment for BDUMP, and visual acuity remained stable without progression. His CMML was managed conservatively with supportive therapy alone, as the disease was in an early, non-proliferative phase and did not meet criteria for cytoreductive or hypomethylating agent therapy at the time of presentation. This management strategy was chosen in coordination with hematology, and no systemic therapy was initiated due to low symptom burden and hematologic stability.

BDUMP can manifest prior to, concurrently with, or after the diagnosis of malignancy. It has been documented as a presenting symptom that led to the discovery of systemic cancer [6,8,9]. In other cases, including the present report, it developed in the context of an already diagnosed malignancy. Bilateral visual symptoms in oncologic patients should raise the suspicion of BDUMP when accompanied by characteristic clinical and imaging features.

Atypical findings such as mucocutaneous involvement or iris lesions have also been reported, broadening the known phenotypic range of this condition [6,9]. Additionally, rectal adenocarcinoma has been identified as an uncommon tumor type associated with BDUMP, further supporting its potential occurrence across diverse malignancies [10].

Although optical coherence tomography (OCT) was not performed in our patient, its diagnostic utility in BDUMP is well-established. OCT can reveal subretinal fluid, outer retinal layer disruption, and RPE alterations, which are valuable for diagnosis and monitoring of disease progression. Kıratlı and Erkan demonstrated these changes clearly in their 2010 report, highlighting the role of OCT in early detection and visual prognosis [11]. In addition, fundus autofluorescence (FAF), which may reveal a characteristic leopard-spot pattern or lipofuscin accumulation, was not performed in this case. The absence of both OCT and FAF represents a limitation in the multimodal assessment and underscores the importance of comprehensive imaging in suspected BDUMP cases.

This case adds to the limited body of literature describing BDUMP in association with hematologic malignancies, and to our knowledge, is among the very few reported in the setting of CMML. It underscores the importance of early recognition, thorough systemic evaluation, and collaborative management between ophthalmologists and hematologists. The case highlights the need to differentiate BDUMP from leukemic infiltration and metastases based on imaging and clinical context, particularly when biopsy is not feasible. While visual acuity remained stable in this case, the prognosis in BDUMP is often guarded, and further research is needed to clarify pathogenesis and develop effective therapeutic strategies.

## 4. Conclusions

BDUMP is a rare paraneoplastic syndrome capable of causing profound vision loss. Although typically associated with solid tumors, hematologic malignancies such as CMML may also be implicated. Prompt recognition, multimodal imaging, and interdisciplinary collaboration are essential. In cases with preserved vision and systemic stability, conservative management may be appropriate. Prognosis remains poor in most cases, and further research is needed to clarify the underlying mechanisms and improve management strategies.

## Data Availability

The original contributions presented in the study are included in the article material, and further inquiries can be directed to the corresponding author.
